# Using CO_2_ to Determine Inhaled Contaminant Volumes and Blower Effectiveness in Several Types of Respirators

**DOI:** 10.1155/2011/402148

**Published:** 2011-07-18

**Authors:** Arthur T. Johnson, Frank C. Koh, William H. Scott, Timothy E. Rehak

**Affiliations:** ^1^Fischell Department of Bioengineering, University of Maryland, College Park, MD 20742, USA; ^2^National Personal Protection Technologies Laboratory, National Institute for Occupational Safety and Health, Pittsburgh, PA, USA

## Abstract

This experiment was conducted to determine how much contaminant could be expected to be inhaled when overbreathing several different types of respirators. These included several tight-fitting and loose-fitting powered air-purifying respirators (PAPRs) and one air-purifying respirator (APR). CO_2_ was used as a tracer gas in the ambient air, and several loose-and tight-fitting respirators were tested on the head form of a breathing machine. CO_2_ concentration in the exhaled breath was monitored as well as CO_2_ concentration in the ambient air. This concentration ratio was able to give a measurement of protection factor, not for the respirator necessarily, but for the wearer. Flow rates in the filter/blower inlet and breathing machine outlet were also monitored, so blower effectiveness (defined as the blower contribution to inhaled air) could also be determined. Wearer protection factors were found to range from 1.1 for the Racal AirMate loose-fitting PAPR to infinity for the 3M Hood, 3M Breath-Easy PAPR, and SE 400 breath-responsive PAPR. Inhaled contaminant volumes depended on tidal volume but ranged from 2.02 L to 0 L for the same respirators, respectively. Blower effectiveness was about 1.0 for tight-fitting APRs, 0.18 for the Racal, and greater than 1.0 for two of the loose-fitting PAPRs. With blower effectiveness greater than 1.0, some blower flow during the exhalation phase contributes to the subsequent inhalation. Results from this experiment point to different ways to measure respirator efficacy.

## 1. Introduction

Development of methods and the determination of inhaled volumes are important for the protection of wearers from airborne contaminants and assignment of minimal expected respirator protection factors [1]. Respirator protection factors are defined as contaminant concentration outside the facepiece divided by contaminant concentration inside the facepiece. These two concentrations are often measured by nondiscriminating particle counters that require a finite amount of time to reach a valid time-averaged measurement. Although this is a relatively simple measurement to make, it cannot be used accurately for rapidly changing particle counts. Particle count is also dependent upon placement within the facepiece, and sharp spatial discontinuities in contaminant concentrations may exist that lead to erroneous conclusions regarding representative facepiece concentrations.

Concentration ratio is only valid as a measure of protection factor as long as there are no particle sources or sinks in the system. It is known that the respiratory system is a source for moisture particles, and these can be counted along with particles of the challenge substance. Protection factors would, in this case, appear lower than they should. Deposition of particles within the respiratory system can also occur as air is inhaled, leading to apparent protection factors higher than they ought to be.

Respirator protection factors may only be an approximate indication of inspiration of contaminants by the wearer. That is because contaminants penetrating the respirator facepiece may not reach the mouth to be inhaled. A more direct indicator of protection afforded by the respirator is wearer protection factor, which we have defined as the concentration of contaminant inhaled divided by ambient concentration. The difference between this and conventional protection factor use is that contaminants inside the respirator facepiece, but not inhaled, are measured conventionally but not for wearer protection factor. 

In addition, a tracer gas, such as CO_2_, can be used to determine the eventual outcome of air supplied by a PAPR blower. This determination we have called blower effectiveness. A blower effectiveness with a value of 1.0 means that all blower air supplied during the inhalation portion of the breathing cycle would contribute to wearer inhaled gas. Some blower air may be lost to the environment directly through the exhalation valve; in this case, blower effectiveness would be calculated as less than 1.0. A blower effectiveness of less than 1.0 would indicate that some inspired air had to come from the ambient, bypassing the blower through respirator leakage. A blower effectiveness greater than 1.0 indicates that some blower air contributed during the exhalation phase would be inhaled in the subsequent inspiration. An APR, without a blower, should have an equivalent blower effectiveness close to 1.0. 

In this study, we investigated measurement of respirator protection factors using a different method of a challenge gas and collection of exhaled breath. It was intended that results from this approach could be a better assessment of protection factors actually experienced by the wearer of the respirator. Because this test used carbon dioxide as the tracer gas, it had to be conducted on a head form, but it did give quantitative assessments of wearer protection factors and blower effectivenesses. The CO_2_ was traceable as originating in the ambient air surrounding the masked head form, and so could help to partition air supplied through the filter from air that bypassed the filter.

## 2. Methods

**Figure 1:**
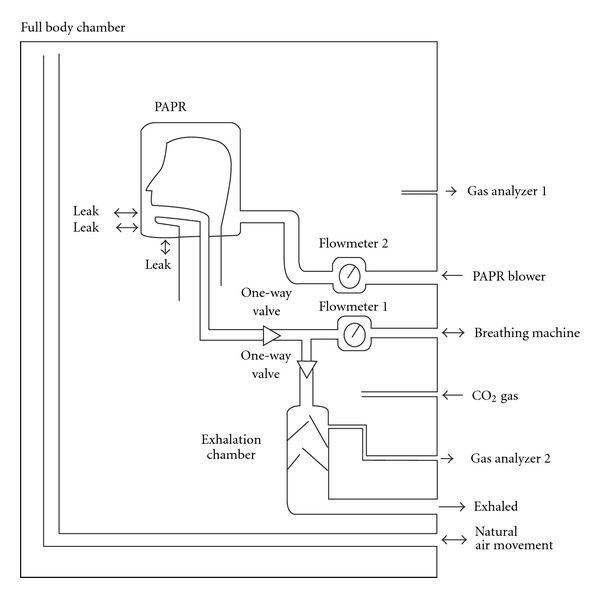
Diagram of the experimental apparatus used. The respirator under test was mounted on a head form inside a large chamber. A breathing machine was used with CO_2_ gas to detect leaks. Exhaled air was collected, and CO_2_ concentration was measured and compared to CO_2_ concentration in the chamber.

Respirator leakage was determined by operating the respirator inside a chamber containing CO_2_ as a tracer gas. Air supply to the respirator or PAPR blower came from the outside atmosphere containing a negligible concentration of the tracer gas. A breathing machine was used to simulate the effect of a human wearer, but exhaled air from the breathing machine was collected in a separate container. The presence of the tracer gas in the exhaled air was quantitative proof of PAPR inward leakage (Figure 1).

The chamber of dimensions 137 cm (54 in) by 76 cm (30 in) by 180 cm (71 in) was constructed of plywood and Lexan transparent plastic for visibility. The plywood was sealed with paint. Placed inside the chamber was a head form on which the respirator was mounted. The mouth of the head form was connected to a breathing machine (Krug Life Sciences, Houston, Tex, USA) outside the chamber by means of a 3.8 cm (1.5 in) flexible ventilator hose (A-M Systems Spiral Tubing, Carlsborg, Wash, USA). Two one-way valves directed inhaled air from the respirator into the breathing machine and exhaled air into a separate container. The valves had been salvaged from U. S. Army M17 air-purifying respirators

The container to collect exhaled breath was constructed from several 2 L soft drink plastic bottles sealed with black electrical tape. The ability of the tracer gas to diffuse through the walls of the container was not investigated, but the short time between breaths, the type of plastic used for the containers, and the volume inside the container made significant concentration errors unlikely.

CO_2_ was used as the tracer gas. The test chamber was filled with 6-7% CO_2_ from a cylinder, and the gas concentration was monitored continuously with a mass spectrometer (Model 1100, Perkin-Elmer, St. Louis, MO). Because CO_2_ inside the test chamber was continuously being replaced with fresh air passing through the respirator, CO_2_ was added continuously from the gas cylinder to maintain the target CO_2_ concentration.

The exhalation gas collection container began with a negligible CO_2_ concentration, which proceeded to climb as exhaled air from more breaths displaced initial air. The air/CO_2_ mixture was sampled continuously by the mass spectrometer and at 50/sec by the data acquisition system. When CO_2_ concentration had reached its final steady-state value, this concentration was used as the value in exhaled air, and, by inference, inhaled air. There was no other place for CO_2_ to go once it was inhaled than into the exhalation collection container.

Respirators tested were the Racal AirMate 3 (Racal, Frederick, Md, USA) loose-fitting PAPR, Breathe Easy (3M, St. Paul, Minn, USA) tight-fitting PAPR, Butyl Head Cover with Cape, #522-02-23 (3M) loose-fitting hood, Centurion MAX (Martindale Protection; Thetford, Norfolk, UK) multipurpose loose-fitting PAPR with scarfs in place, SE 400 (SEA, Meadowlands, Pa, USA) breath-responsive PAPR, and FRM 40 (3M) air-purifying respirator. MedGraphics (St. Paul, Minn, USA) #5038773 pitot tube flowmeters were used to measure blower flow and breathing machine flow. These were carefully calibrated beforehand to ensure that they gave identical measurements for identical flow rates. Flowmeters were adapted to blower inlets for the Racal, Centurion, and SEA devices. Flowmeters were placed in the connecting hose between blower and facepiece for the two 3M powered devices. An inlet hose was connected to the filter of the FRM40.

PAPR blowers were operated with fully charged batteries, and each test lasted approximately 2 min. Hoses were attached to the inlets of each blower so that ambient air could be drawn from outside the chamber. Hose inlets were located about 1 m above the chamber, and excess gas was exhausted from the chamber floor in order not to cycle CO_2_ from the chamber back into the respirator inlets (CO_2_ is denser than air). 

The breathing machine was set to generate a minute volume of 112 L/min, tidal volume of 2.4 L, and a peak flow of 317 L/min. The breathing wave shape was sinusoidal. These settings have been used in this and previous experiments in order to induce respirator leakage if the respirator is going to leak at all. The breathing machine minute volume is nearly the same as most PAPR blower flow rates, but peak flows are much higher than blower flow rates.

Data were collected with an analog-to-digital data acquisition board (National Instruments, Austin, Tex, USA) connected to the Universal Serial Bus (USB) of a PC computer. Custom software developed in LabView 7 (National Instruments, Austin, Tex, USA) recorded flow and concentration data, calculated flow differences, and exhaled volumes.

The volume of inhaled CO_2_ is the leakage volume times the concentration of CO_2_ in the exhalation collection chamber atmosphere, which also equals the exhaled volume times the exhaled CO_2_ concentration. Thus, leakage volume can be obtained as the exhaled volume times the ratio of CO_2_ concentrations in the exhaled breath and chamber atmosphere.

## 3. Results

Results are summarized in Table 1. The leftmost column includes values obtained from the readings ($\dot{V}$) of flowmeter #2 in Figure 1. Where there is a range of values, the blower flow rate varied throughout the breathing cycle. Inhaled volume (*V*_inh_) was obtained by integrating the flow signal (${\dot{V}}_{\text{inh}}$) from flowmeter #1 during the inhalation portion of the breathing cycle. Exhaled volume (*V*_exh_) was obtained by integrating the flow signal (${\dot{V}}_{\text{exh}}$) from flowmeter #1 during the exhalation portion of the breathing cycle. Normally, both Inhaled volume and exhaled volume would have been expected to agree within the error rate of the measurement and integration process. There is a larger than expected difference of up to 10% between the two volumes, possibly attributed to some cooling of the air as it resided in the breathing machine or compression of the exhaled air as it left the breathing machine. Because of the differences between to two volumes, the appropriate volume was chosen to calculate additional derived values.

The CO_2_ ratio in Table 1 is the ratio of CO_2_ concentration measured in the captured exhaled air (*C*_CO_2_,exh_) divided by the CO_2_ concentration in the ambient air in the chamber surrounding the respirator (*C*_CO_2_,amb_). The wearer protection factor was calculated as the inverse of the CO_2_ ratio, (*C*_CO_2_,amb_/*C*_CO_2_,exh_). 

Leakage volume (*V*_leak_) represents the volume of air that leaked into the respirator facepiece and was subsequently inhaled by the breathing machine. Leakage volume was calculated as the exhaled volume times the CO_2_ ratio. This came from a CO_2_ mass balance on the respirator:

(1) $$\begin{array}{cc} & \text{Amount}\;\text{of}\;{\text{CO}}_{2}\;\text{in}-\text{Amount}\;\text{of}\;{\text{CO}}_{2}\;\text{out}+{\text{CO}}_{2}\;\text{generated}\\ & \;\;=\text{Amount}\;\text{of}\;{\text{CO}}_{2}\;\text{stored}. \end{array}$$

The amount of CO_2_ into the respirator was the net leakage volume times the CO_2_ concentration in the ambient air in the chamber surrounding the respirator. Any leakage of air and CO_2_ out of the respirator was already included in the net leakage of air containing CO_2_ into the respirator. There was no CO_2_ generated in this apparatus. The amount of CO_2_ stored was the amount of CO_2_ collected in the container positioned to accumulate exhaled air and equaled the exhaled volume times the CO_2_ concentration in the collected exhaled volume. Thus, (1) became

(2) $$\begin{array}{c} V_{\text{leak}}\left(C_{{\text{CO}}_{2},\text{amb}}\right)+0+0=V_{\text{exh}}\left(C_{{\text{CO}}_{2},\text{exh}}\right),\\ V_{\text{leak}}=V_{\text{exh}}\left(\frac{C_{{\text{CO}}_{2},\text{exh}}}{C_{{\text{CO}}_{2},\text{amb}}}\right). \end{array}$$

The blower contribution to the inhaled volume was calculated from

(3) $$V_{\text{bl},\text{inh}}=V_{\text{inh}}\left[1-\frac{C_{{\text{CO}}_{2},\text{inh}}}{C_{{\text{CO}}_{2},\text{amb}}}\right].$$

This equation indicates that the blower air volume contribution (*V*_bl,inh_) to total inhaled volume (*V*_inh_) is the proportion of air not identified as leakage (1 − *C*_CO_2_,inh_/*C*_CO_2_,amb_). 

The total volume of filtered air passing through the blower during the time for inhalation, labeled total blower volume (*V*_bl,tot_) in Table 1, can be obtained from blower flow rate ($\dot{V_{\text{bl}}}$) measured by flowmeter #2 in Figure 1, integrated over the total inhalation time (*t*_inh_).

Blower effectiveness is the ratio of the blower contribution to inhaled air (*V*_bl,inh_) to total blower volume (*V*_bl,tot_) during the inhalation portion of the breathing cycle

(4) $$\text{Eff}=\frac{V_{\text{bl},\text{inh}}}{V_{\text{bl},\text{tot}}}.$$

**Figure 2:**
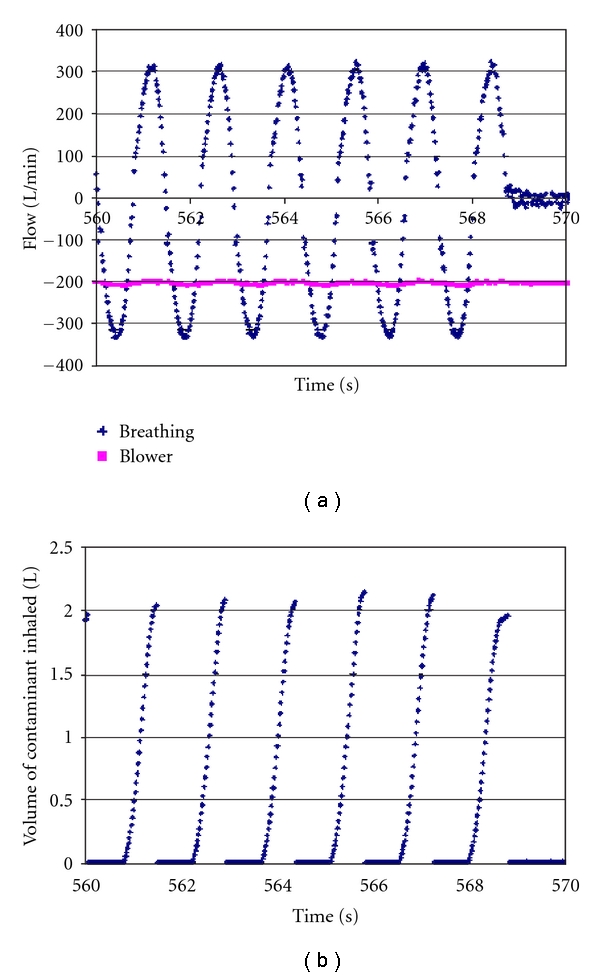
Flows and volumes for the Racal AirMate 3 loose-fitting PAPR. Breathing machine flow was sinusoidal with exhalation in the positive direction. Blower flow rate changed hardly at all. Corresponding contaminant volumes were calculated as the CO_2_ concentration in the exhaled air times the integral of the breathing machine exhalation flow rate.

In Figure 2 are shown breathing machine flow rate, blower flow rate, and inhaled leakage volumes (labeled inhaled contaminant volumes) for the Racal AirMate 3 loose-fitting PAPR. This figure is intended to show that blower flow rate for this respirator is almost constant, and that inhaled contaminant volume (integrated breathing machine flow rate times CO_2_ concentration in the captured exhaled breath) is slightly more than 2 L for a breathing machine tidal volume of 2.4 L.

**Figure 3:**
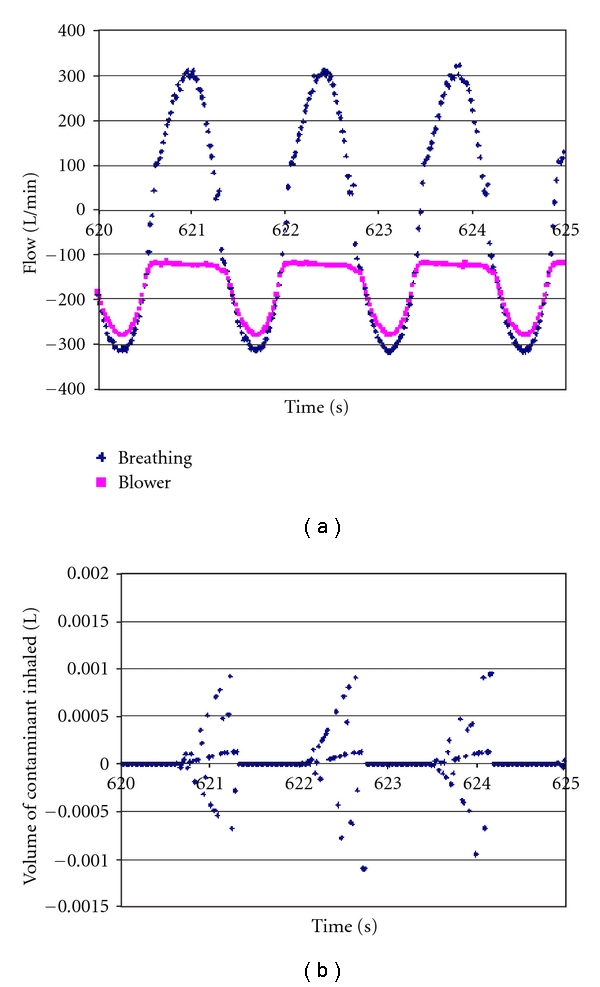
Flows and volumes for the 3M Breathe Easy tight-fitting PAPR. Blower flow rate can be seen to track breathing machine flow during inhalation. The corresponding contaminant volumes (below) were so small that they were inconsequential.

Figure 3 is similar to Figure 2 but shows that blower flow rate for the 3M Breathe Easy tight-fitting PAPR varies throughout the inhalation phase of breathing. The volume of inhaled contaminants is indistinguishable from zero.

**Figure 4:**
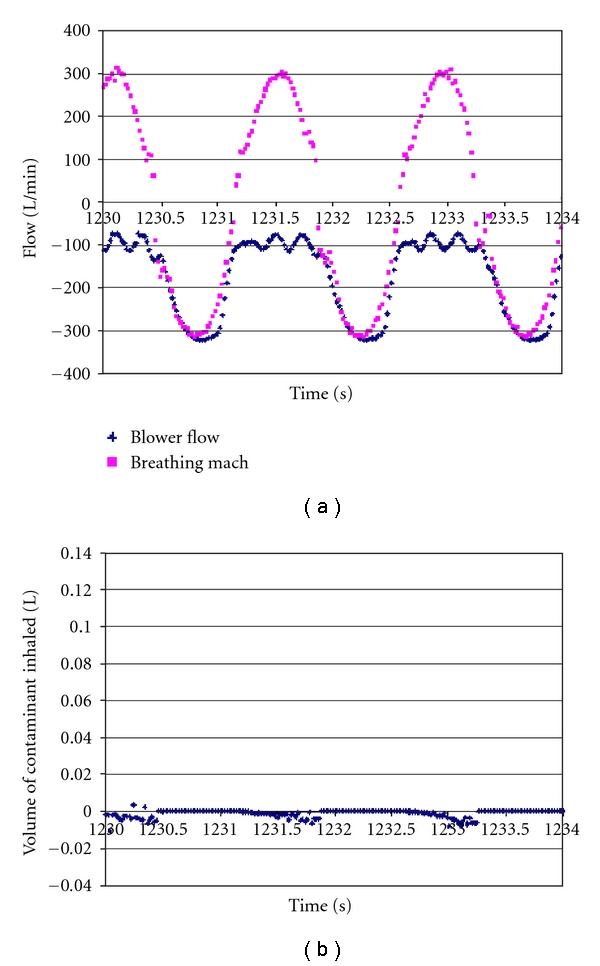
Flows and volumes for the SE 400 breath-responsive PAPR. Blower flow rate was adjusted to maintain positive pressure inside the face piece. Corresponding contaminant volumes (below) were negligible.

Figure 4 illustrates responses by the SE 400 breath-responsive tight-fitting PAPR. Blower flow rate tracks breathing machine flow rate in an attempt to maintain positive pressure inside the facepiece. Again, inhaled volume of contaminants is zero.

## 4. Discussion

It has been seen previously in our lab that PAPR blower flow rates vary during the breathing cycle, and this is also reflected in the entries for blower flow rates. Exhaled tidal volumes were nearly constant at 2.32 to 2.42 L. The ratio of CO_2_ concentrations in the exhaled breath and enclosing chamber is also shown. The inverses of these figures are the measured protection factors for each of the respirators as worn and used. In the column labeled “Leakage Volume” are found volumes of inhaled contaminant-laden air, obtained by multiplying the concentration ratios by exhaled volumes. These figures also represent nominal leakage volumes for the respirators.

Despite advances in filter technology, contaminant levels inside respirators can still become unacceptably high if the respirators can leak ambient air through alternate pathways. In fact, weak links in wearer protection are the facial fit and the exhalation valve. The figure of merit for respiratory protection has been the protection factor (PF) defined as the concentration of contaminant outside the respirator divided by the concentration inside the respirator.

In this study, we used an alternative means to measure protection factor as it is related to respirator leakage. If the respirator was operated in an atmosphere containing a tracer gas, but the supply of air to the respirator through the filter circuit was free of tracer gas, then the only means for the gas to enter the facepiece and be inhaled was if the gas leaked inward from some path different from the filter circuit. If this test was conducted with a breathing machine, then the tracer gas would neither be deposited nor absorbed in the machine. The average concentration of the gas in the collected exhaled breath should then be equal to the average concentration as presented to the mouth inside the facepiece, thus averaging regions of high and low concentrations due to preferred contaminant flow pathways. The ratio of tracer gas concentration in the collected exhaled breath to the gas concentration in the surrounding atmosphere should then be the inverse of PF, at least from the wearer's standpoint.

It can be noted that, although the Racal AirMate 3 blower flow rate was higher than the Centurion MAX blower flow rate, contaminant exposure with the Racal is much higher and protection factor is much lower. With a protection factor nearly equal to 1.0, the Racal loose-fitting PAPR gives almost no protection against airborne contamination; concentration of contaminant inside the respirator shield is nearly the same as outside the shield. The Centurion MAX loose-fitting respirator gives somewhat better protection, but still not enough to be very effective.

The SE 400 breath-responsive respirator attempts to maintain positive pressure inside the facepiece. The SE 400 blower flow can be seen to exceed the peak flow of 317 L/min produced by the breathing machine. When the blower was turned off, flow through the blower was much lower, and the respirator operated as an air-purifying respirator (APR). The FRM 40 APR has a similar flow rate through its filter, but a somewhat lower protection factor.

Three of the respirators tested had no evidence of contamination in the exhaled air. These were the 3M Hood, 3M PAPR, and SE 400 PAPR. In very demanding environments, these respirators would afford the best protection. Even if the power fails on the SE 400, measured protection factor is still 20. The 3M Hood was, in effect, a loose-fitting respirator, yet had enough dead volume within its enclosure that contaminants did not reach the mouth.

Results from this study are not totally in agreement with some of the Assigned Protection Factors published by OSHA [1]. OSHA assigns a protection factor of 50 to a full face piece APR. Our results for the FRM 40 give a protection factor of 17, and for the unpowered SE 400 a value of 20. The OSHA value for full-face piece tight-fitting PAPR is 1000; our results for the 3M PAPR and powered SE 400 are extremely high, infinite in our tests. The OSHA value for the loose-fitting PAPR is 25; we obtained values of 1.1 for the Racal, 4 for the Centurion, and infinity for the 3M Hood. 

There are two difficulties with PF as currently measured. First is that contaminant concentration inside the respirator can be nonuniform, and, thus, the measurement can be dependent upon location. Recent studies on flow visualization inside respirator facepieces [1] have shown that flow pathways can twist and curl, with clear delineation between contaminant-filled air and clean air over very short distances. Placing a contaminant-detection probe in a stagnant zone could yield measurements that probably underestimate contaminant concentration. Placing the probe in the flow pathway, where contaminant concentration might be particularly high, could overestimate average concentration in the facepiece. Present practice is to place the probe in front of the mouth; this somewhat corrects the placement problem, because this is the place where inhaled air is to be drawn but still does not solve the problem of time variation in contaminant levels at the place where they are likely to be inhaled.

The second difficulty with PF is that some contaminants can deposit or be absorbed in the respiratory system, thus making the respiratory airways into a contaminant filter. Measured average containment concentration inside the facepiece would, therefore, be underestimated. Thus, it may not be surprising that results obtained in this study did not match those obtained with other methods.

Dead volume within a respirator facepiece is often thought to be detrimental because it accumulates exhaled CO_2_ and recycles it into the next inhaled breath and limiting physical work performance [2]. Dead volume inside the facepiece, however, can be helpful if it acts as a buffer against leaked contaminants reaching the mouth. Dead volume can be protective especially if air during the exhalation phase of breathing purges the enclosed air of contaminants leaked during inhalation. This can happen with PAPR blowers, for instance, when they supply filtered air even during exhalation. Protective dead volumes of some of these respirators had previously been measured. For the Centurion MAX, that value was about 1.4 L, and for the Racal AirMate, it was close to zero. Dead volume of the FRM 40 is about 1.0 L, and the other two tight-fitting facepieces were presumed to have about the same amount. The protective dead volume of the 3M Hood was measured on a mannequin by connecting the inlet port at the mannequin mouth to a vacuum hose and the inlet hose from the blower was closed off. The mannequin with Hood was placed inside a fog-filled chamber. Mouth flow was recorded for the length of time for the fog to reach the mouth. As expected, fog entered the facepiece from below the ear and chin along the neck. The total volume of air inhaled before the fog reached the mouth was 2 L.

How large should protective dead volume be? It could be made large enough so that no contaminated air would reach the mouth even under extreme circumstances. Inhaled tidal volumes during physical exertion are normally in the 1.5 L range, sometimes reach 2.0 L, and only rarely exceed 2.5 L. Protective dead volumes greater than 2.5–3.0 L should then be at least as effective as continuous positive pressure in the face piece in providing wearer protection, as long as the blower can purge the dead volume during the exhalation phase of the breathing cycle. Dead volumes larger than 2.5–3.0 L require larger blower flow rates and may be unnecessary and undesirable. 

Comparing results from this and other recent fog flow visualization studies shows that protective respirator dead volume measurements are generally in agreement no matter what procedure is used. Using flow visualization with a breathing machine gave an inhaled volume before fog reached the mouth of about 1.4 L for the Centurion MAX PAPR. Flow visualization with human subject breathing gave 1.1 L before fog reached the mouth for the same respirator [3].

It is known that peak respiratory flows can often exceed blower flows [2, 4–9]. There has also been concern expressed when the pressure inside a respirator facepiece intended to be positive pressure that becomes negative momentarily [10]. As long as positive pressure is maintained, it is asserted, any leakage would flow from inside the facepiece to the outside, and contaminated air would not enter the facepiece. If PAPR blowers and batteries were made powerful enough to perform up to peak flow levels, bulk of the devices would probably increase greatly, and the extra weight could reduce work performance [11]. An alternate strategy, one that has just recently been realized, is to ensure a large enough protective dead volume that any contaminants entering the facepiece do not reach the mouth. Without accounting for exhalation air coming from the mouth, blower flow rate does not need to be any larger than that required to purge the dead volume of contaminants during the exhalation phase, so long as the distribution of blower air is wide enough to sweep the entire dead volume. Taking exhaled air into account reduces required blower flow rate even further, at least as far as contaminants are concerned. Smaller blower flow rates might result in CO_2_ accumulation in the dead volume. The net result is that peak inhalation flow rates do not have to be met by the blower as long as stored clean air is available inside the facepiece.

Nevertheless, results in this and other recent experiments have shown that it is not necessary to maintain positive pressure inside the facepiece at all times just as long as contaminated (leakage) air never reaches the mouth. The blower must be able to remove contaminated air from the protective dead volume before the next negative pressure incident.

Many of the newer hood-type loose-fitting respirators use the protective dead volume principle, and so can be considered at least as protective as APRs and tight-fitting PAPRs. They have the advantage over APRs in that there is no high resistance to breathe through but have the disadvantage compared to tight-fitting PAPRs that, should the blower be inoperable, there is no contaminant protection afforded.

The validity of the methods used in this study depends upon filter efficacy. The tracer gas used in this study was CO_2_, and we did not allow CO_2_ to challenge the filter; each filter inlet was supplied by clean air. Hence, perfect filter efficiency was assumed, and we were mainly interested in respirator leakage.

If a different gas was used, one that the filter should remove, then the inlet to the filter could be in contact with the same atmosphere that surrounds the respirator under test. That way the filter circuit would also be tested.

Clayton et al. [12] calculated respirator protection factors for human wearers while they simulated asbestos removal operations. They used a method similar to that used in the present study, except that their subjects worked in a chamber containing a small concentration of sodium hexafluoride instead of carbon dioxide. They also continuously measured SF_6_ concentrations inside and outside the respirator, and thus, could measure protection factors as they varied throughout the breathing cycle. In the present study, we were more interested in knowing how much of the contaminated air was actually inhaled, so inhaled contamination (CO_2_) was collected when it was expelled from the breathing machine. Obviously, CO_2_ could not be used as a test gas with human test subjects; SF_6_ or CH_4_ might be a better choice. However, collecting exhaled gas and determining contaminant levels there rather than monitoring contaminant levels inside the respirator facepiece give the actual protection factor experienced by the wearer as compared to the respirator protection factor (insofar as there is no gas absorbed in the respiratory system).

The average concentration of tracer gas in the exhaled breath would not be expected to equal the average concentration inside the facepiece. Rather, the average exhaled-breath concentration reflects the concentration of contaminant actually inspired. This means that regions of high flow leading to the mouth are weighted substantially more than regions of nearly-stagnant flow. In this respect, measurement of exhaled-breath concentration is an honest measure of the exposure of the wearer.

Reports have been published relating facial measurements of wearers to respirator fit [13]. One reason for this, of course, is that some facial configurations result in large leaks, and thus, lower protection factors. However, results from this study indicate another possible cause, and that is flow pathway of contaminated air. Different facial configurations could channel leakage flows differently in different people. Depending on the exact position of the particle counter used in those studies [13–15], the counter could register higher average values or lower average values when contaminants were drawn into the respirator facepiece. Although several of these studies were conducted with half facepiece or filtering facepiece respirators [13–17], there is no reason to suspect that preferential flow pathways discovered in studies with loose-fitting PAPRs or tight-fitting APRs are not also present in other types of respirators. Facial configuration, especially nose protrusion, could easily affect leakage flow pathway to the mouth.

Calculation of net overbreathed volume as the integral of the difference between mouth flow and blower flow depends on the assumption that all the blower flow is captured within the facepiece. Likewise, the statement made earlier that the blower needed to supply a flow rate no larger than the facepiece dead volume divided by the exhalation time is contingent upon no blower flow escaping the face piece before it sweeps the facepiece. It is likely that some blower flow escapes directly to the outside, either through leaks or through the exhalation valve. This represents inefficient use of blower capacity. At present, there are no known published measurements of ineffectual blower flow, but these measurements are able to be made with the same method as used in this experiment.

If contaminated air can leak from outside the facepiece into the inhaled breath, then blower air can flow directly out of the facepiece without contributing to clean air in the protective dead volume. 

It can be seen that blower effectiveness is very low for the Racal respirator. This indicates that most of the air propelled by the blower does not contribute to the volume of air inhaled and is consistent with other data relating to protection factor in this study and previous studies [18].

The 3M Breathe Easy tight-fitting PAPR has a blower effectiveness of about 1.0, which indicates that nearly all of the blower flow contributes to inhalation. The same is true for the SE 400 with blower turned off and FRM 40 APR; nearly all the air flowing through the blower or filter pathway contributes to inhaled air.

When the SE 400 blower was turned on, positive pressure is maintained in the facepiece at least most of the time, and some of the blower air leaks out, probably through the exhalation valve. Blower effectiveness for this PAPR is about 90%. Ten percent of the air supplied by the blower is wasted to the atmosphere.

Blower effectivenesses for both the Centurion MAX and 3M hood were greater than 1.0. In both of these cases, there apparently was enough protective dead volume that air supplied by the blowers during the exhalation phase of breathing contributed to inhaled volume. In these two cases, blowers conform to the recommendation we made that the blower need not supply all inhaled air volume but it does need to purge dead volume air during the exhalation interval. Thus, the blower can contribute to inhaled air volume even during exhalation, although data in Table 1 indicates that not all contaminant is evacuated.

Blower effectiveness analysis is only approximate at this time. The flow situation inside the respirator face piece is complex, involving air leaking in as well as out simultaneously. Dead volume within the face piece accumulates air from all sources overtime. Breathing is periodic and not steady, and flow pathways inside the face piece most likely change between inhalation and exhalation, and probably over time within breathing phase as well. If significant physical movement accompanies work performance, then the respirator can shift on the face, or in the case of the hood, the volume of air inside the hood covering the torso can change greatly. Under extremely difficult breathing conditions, the facepiece itself can deform, which changes the boundary of the flow domain. At this point, successful determination of flow dynamics within a respirator has not been accomplished, to our knowledge.

## 5. Conclusions

These results demonstrated that there was a large range of wearer protection factors among different types of respirators. Some of the respirators tested provided little to no protection to the wearer, whereas others provided extremely high amounts of protection. The results also showed that blowers have multiple contributions to PAPR performance, including providing inhalation air, cleaning contaminant from the respirator facepiece, and perhaps adding to PAPR leakage. The concept of blower effectiveness was introduced as a way to rate the ability of the blower to supply clean inhalation air to the wearer.

## Figures and Tables

**Table 1:** Summary of results.

Respirator	Blower flow rate (L/min)	Exhaled volume (L)	CO_2_ ratio	Wearer protection factor	Leakage volume (L)	Inhaled volume (L)	Blower contribution (L)	Total blower volume (L)	Blower effectiveness
Racal PAPR	191–200	2.41	0.84	1.2	2.02	2.66	0.43	2.42	0.18
Centurion PAPR	88–101	2.37	0.25	4	0.60	2.66	1.99	1.17	1.70
3M hood PAPR	157–161	2.39	0	*∞*	0	2.63	2.63	1.87	1.41
3M PAPR	121–278	2.42	0	*∞*	0	2.62	2.62	2.51	1.04
SE 400 PAPR	64–322	2.32	0	*∞*	0	2.58	2.58	2.90	0.89
SE 400 APR (blower off)	(0–284)	2.37	0.048	21	0.11	2.58	2.46	2.50	0.98
FRM 40 APR	0–289	2.37	0.057	18	0.14	2.62	2.47	2.51	0.99
